# Evaluation of hemostasis understanding in medical and pharmacy students from a Parisian university

**DOI:** 10.1016/j.rpth.2024.102547

**Published:** 2024-08-22

**Authors:** Nicolas Gendron, Dominique Helley, Philippe Rousselot, Virginie Siguret, Pascale Gaussem, Chloé James, Lina Khider, Nadine Ajzenberg, Elodie Boissier, Nicolas Boissel, David M. Smadja, Benjamin Planquette

**Affiliations:** 1 Paris Cité University, Innovative Therapies in Haemostasis, INSERM, Paris, France; 2 Hematology Department, Assistance Publique Hôpitaux de Paris, Centre-Université de Paris (APHP-CUP), Paris, France; 3 F-CRIN INNOVTE, Saint-Étienne, France; 4 Paris Cité University, INSERM, PARCC, Paris, France; 5 Hematology Department, Centre Hospitalier de Versailles, Université Versailles Paris-Saclay, Versailles, France; 6 Hematology Department, AP-HP, Hôpital Lariboisière, Paris, France; 7 Laboratory of Hematology, University Hospital, Bordeaux, France; 8 University of Bordeaux, INSERM UMR 1034, Biology of Cardiovascular Diseases, Pessac, France; 9 Department of Vascular Medicine, Assistance Publique Hôpitaux de Paris, Centre-Université de Paris (APHP-CUP), Paris, France; 10 Paris Cité University, Laboratory of Vascular Translational Science, INSERM, Paris, France; 11 Laboratoire d'Hématologie, AH-HP, Bichat–Claude Bernard Hospital, Paris, France; 12 Laboratory of Hematology, University Hospital, Nantes, France; 13 Paris Cité University, Hematology Adolescent and Young Adult Unit, Saint-Louis Hospital, AP-HP, URP-3518, Institut de Recherche Saint-Louis, Paris, France; 14 Respiratory Medicine Department, Assistance Publique - Hôpitaux de Paris-Centre (APHP-CUP), Paris, France

Hemostasis is complex and concerns several medical fields, presenting a considerable challenge for medical doctor (MD) students to assimilate. Insufficient knowledge regarding coagulation and its associated tests has led to widespread inappropriate ordering and erroneous interpretation [[1], [2], [3]]. Laboratory testing is crucial for clinical decision-making [4]. Inappropriate hematology laboratory testing is common [[5], [6], [7]]. The mean rate of inappropriate overuse was 20.6% for all laboratory tests and 33.3% for hematology tests, including hemostasis panels [7]. During the COVID-19 pandemic, we observed a significant increase in D-dimer assays due to COVID-19–associated coagulopathy. This was also seen with hemograms and antiplatelet factor 4 heparin antibodies [8], suggesting overtesting likely due to concerns about vaccine-related thrombosis and misinformation/disinformation. Previous studies showed increased laboratory testing in teaching hospitals compared with nonteaching hospitals [9], possibly due to the training environment. A Canadian study showed that resident physicians and MD students had poor knowledge of routine hemostasis tests [1].

Overprescribing tests leads to unnecessary expenses and potential patient harm [10,11]. In France, medical advice on hemostasis, often provided by clinical pathologists specializing in hematology, is frequently requested. An unpublished survey by the French Society on Thrombosis and Hemostasis reported that 31% of clinical pathologists provide between 5 and 10 hemostasis-related medical advice daily. Clinical pathologists can be MD or pharmacist (PharmD) in France, Belgium, and Switzerland.

The objective of this study was to assess hemostasis knowledge among MD and PharmD students at one of the largest French universities and identify potential knowledge gaps. We designed a 10-point hemostasis knowledge quiz with 5 multiple-choice questions (MCQs) and 5 short-answer open-ended questions (Supplementary Methods) covering various hemostasis aspects relevant to clinical practice, in line with the French MD student national program.

Our study included i) MD students from Paris Cité University (UPC) Medical School completing the fourth, fifth, and sixth years; ii) PharmD students of UPC Pharmaceutical School completing the third and fourth years; and 3 control groups: iii) all French hematologist residents specialized in malignant and classical hematology, including hemostasis physiopathology during their training; iv) members of the French Club of Young Vascular Physicians from the French Society of Vascular Medicine specialized in thrombosis and anticoagulation; and v) hemostasis consultants from the French Society on Thrombosis and Hemostasis, serving as a positive control for hemostasis knowledge.

The study adhered to the Helsinki Declaration and was approved by the education council and review board of the medical and pharmaceutical schools of UPC. Continuous data were expressed as median (IQR) or mean ± SD and categorical data as proportions. Statistical analyses were performed using the Mann–Whitney U-test or Kruskal–Wallis test, followed by Dunn’s multiple comparisons test using Prism (version 9.0; GraphPad). All analyses were 2-sided, with a *P* value of <.05 considered statistically significant.

Between April 14 and June 20, 2022, we obtained responses from 163 MD and 90 PharmD students, 43 hematologist residents, 22 vascular physicians, and 69 hemostasis consultants (Supplementary Figure S1). Demographic characteristics are described in the Table. Clinical rotation in a general laboratory of hematology was realized for 7 (4.3%) MD students and 18 (41.9%) hematologist residents. However, only 6 (14.0%) completed a rotation in a laboratory specialized in hemostasis. Among hemostasis consultants, 36 (52.2%) were MD and 33 (47.8%) were PharmD practicing as clinical pathologists.

**Table:** Demographic characteristics of the participants and hemostasis knowledge scores.

	MD students *n* = 163	PharmD students *n* = 90	Hematologist residents *n* = 43	Vascular physicians *n* = 22	Hemostasis consultants *n* = 69
Age (y), median (IQR)^a^	23.0 (22.0-24.0)	21.0 (20.0-22.3)	28.0 (25.0-30.0)	34.0 (30.8-36.0)	48.0 (40.0-58.0)
Gender, *n* (%)					
Men	51 (31.3)	27 (30.0)	20 (46.5)	14 (63.5)	16 (23.2)
Women	110 (67.5)	62 (68.9)	22 (51.2)	8 (36.4)	51 (73.9)
Nonbinary	1 (0.6)	1 (1.1)	0 (0.0)	0 (0.0)	0 (0.0)
NA	1 (0.6)	0 (0.0)	1 (2.3)	0 (0.0)	2 (2.9)
Student or resident training, *n* (%)					
Internal medicine	44 (27.0)	NA	20 (46.5)	11 (50.0)	NA
Cardiology	57 (35.0)	NA	0 (0.0)	16 (72.7)	NA
Vascular medicine	12 (7.4)	NA	0 (0.0)	NA	NA
Intensive care unit	107 (65.6)	NA	24 (55.8)	5 (22.7)	
Hematology	43 (26.4)	NA	43 (100.0)	0 (0.0)	NA
Laboratory of hematology	7 (4.3)	NA	18 (41.9)^b^/6 (14.0)^c^	0 (0.0)	NA
Year of medical/pharmaceutic school, *n* (%)					
Third	0 (0.0)	40 (52.2)			
Fourth	53 (32.5)	43 (47.8)			
Fifth	70 (42.9)	0 (0.0)			
Sixth	40 (24.5)	0 (0.0)			
Years since graduation, median (IQR)^a^	NA	NA		2.5 (0.0-5.0)	12.0 (6.5-22.0)
MD, *n* (%)				22 (100.0)	36 (52.2)
PharmD, *n* (%)				0 (0.0)	33 (47.8)

MD, medical doctor; NA, not available; PharmD, pharmacist.

a Interquartile 25th to 75th percentiles.

b Laboratory specialized in hematology malignancies.

c Laboratory specialized in hemostasis.

**Figure 1:**
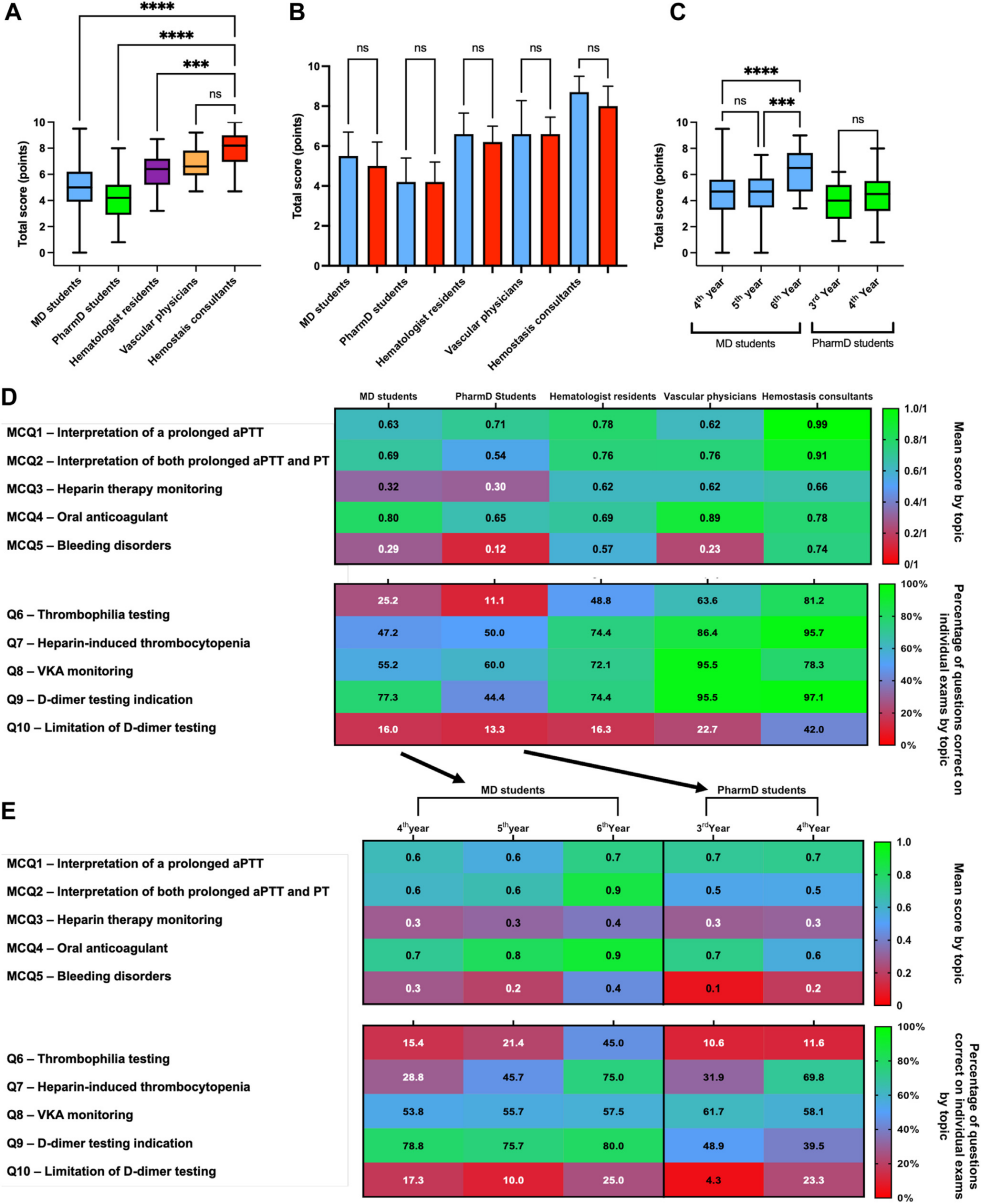
Hemostasis knowledge quiz score. (A) Comparison of hemostasis knowledge quiz scores between groups and hemostasis consultants. Medical doctor (MD) students (4.9 ± 1.8), pharmacist (PharmD) students (4.1 ± 1.5), hematologist residents (6.3 ± 1.4), vascular physicians (6.7 ± 1.3), and hemostasis consultants **(**8.0 ± 1.4). (B) Comparison of hemostasis knowledge quiz score according to gender (blue for men and red for women). (C) Hemostasis knowledge quiz score according to students’ years of medical and pharmaceutical studies. MD students in fourth (4.4 ± 1.8), fifth (4.6 ± 1.6), and sixth year (6.1 ± 1.6); PharmD students in third (3.9 ± 1.4) and fourth (4.3 ± 1.6; *P* = .18) year. (D) Mean scores for individual multiple-choice questions (MCQs) and percentages of correct answers for individual short open-ended questions (Qs) analyzed by topic in each group. (E) Mean scores for individual MCQs and percentages of correct answers for individual Qs analyzed by topic and according to students’ years of medical and pharmaceutical studies. ∗∗∗: *P*-value < .001; ∗∗∗∗: *P*-value < .0001. aPTT, activated partial thromboplastin time; ns, not significant; PT, prothrombin time; VKA, vitamin K antagonist.

Hemostasis consultants had the highest quiz scores (8.0 ± 1.4), significantly higher than MD students (4.9 ± 1.8), PharmD students (4.1 ± 1.5), and hematologist residents (6.3 ± 1.4; *P* < .001 for each; Figure 1A). No significant difference was found between MD and PharmD students’ scores (*P* = .053), nor between hemostasis consultants with MD or PharmD diplomas (7.9 ± 1.3 vs 8.1 ± 1.6; *P* = .45). Gender did not significantly affect scores (Figure 1B). MD students’ scores improved significantly by the sixth year (6.1 ± 1.6) compared with the fourth and fifth years (4.4 ± 1.8 and 4.6 ± 1.6, respectively; *P* < .001 for each; Figure 1C). PharmD students showed no significant score differences between the third and fourth years. Hematologist residents’ score did not correlate with residency duration (Supplementary Figure S2).

We analyzed the mean score for individual MCQs and the percentage of correct answers for individual short open-ended questions by topic (Q). Hemostasis consultants excelled in most topics except oral anticoagulation and vitamin K antagonist monitoring, where vascular physicians performed better (Figure 1D). Scores for heparin therapy monitoring (Q3) were low among MD and PharmD students, while hematologist residents showed superior knowledge in bleeding disorders (Q5), thrombophilia testing (Q6), and heparin monitoring (Q3). For D-dimer testing (Q9), PharmD students had the lowest correct response rate (44.4%), while other groups scored above 70%. Concerning D-dimer testing limitation (Q10), the percentage of questions correct was very low in MD and PharmD students and also in hematologist residents (16.0%, 13.3%, and 16.3%, respectively) and low in vascular physicians (22.7%) and only at 42.0% for hemostasis consultants.

We analyzed the scores for individual MCQs by topic and year among MD and PharmD students (Figure 1E): sixth-year MD students showed significant improvements in thrombophilia testing, heparin-induced thrombocytopenia (Q7), and D-dimer testing limitations. PharmD students showed improvement only in heparin-induced thrombocytopenia and D-dimer testing limitations.

MD students had fewer hours of hemostasis teaching compared with PharmD students (13.28 vs 23.25 hours; Supplementary Figure S3). Only MD students have on-demand virtual classes, which differs from PharmD students.

Our study observed lower hemostasis knowledge in MD and PharmD students from UPC compared with hemostasis consultants. However, MD students' knowledge improved over the years, while PharmD students’ knowledge did not. Hemostasis testing, which includes coagulation tests, thrombophilia testing, and D-dimer testing, is poorly understood by MD students, residents, and clinicians and is often misordered [5,12]. MD and PharmD students, future senior physicians, and pathologists showed low knowledge in key hemostasis areas: bleeding disorders, heparin monitoring, thrombophilia testing, and limitation of D-dimer testing. In contrast, MD students demonstrated good knowledge of oral anticoagulation. After reviewing the answers to the question about the situation (under anticoagulant therapy) where D-dimer is not interpretable for the diagnosis of venous thromboembolism [13], we were surprised by the low percentage of correct responses across all groups. Similarly, low correct responses in heparin monitoring were observed even among experienced clinicians. However, in clinical practice, numerous hemostasis advice are given daily regarding heparin monitoring. Although there is some overlap in scores between medical students and hematology residents, the latter demonstrated superior knowledge in bleeding disorders, thrombophilia testing, and heparin monitoring. This superior understanding of heparin monitoring among residents could be attributed to their daily management of patients and anticoagulation therapies in the hospital, unlike MD students, who bear less responsibility.

In our study, the hemostasis knowledge score was not different between MD and PharmD students but PharmD students had more hours of hemostasis teaching. We acknowledge that PharmD and MD students are different and have a different global formation program. Indeed, since third year of formation, MD students are in hospital every day for clinical rotation that could be practice learning. PharmD students realized clinical rotation only during the fourth year. This could explain the higher knowledge quiz scores among sixth-year MD students, but it might also be due to the fact that the quiz was sent one month before the national ranking exams during their intensive revision period.

Gabarin et al. [1] conducted a survey in internal medicine resident physicians and identified several key issues: (i) trainees reported difficulties in understanding coagulation due to traditional pedagogical approaches; (ii) historical hemostasis teaching uses Roman numerals and focuses on the coagulation cascade, which is challenging and does not accurately reflect physiological processes *in vivo*; (iii) there was a gap between theoretical teaching and practical application; and (iv) there was a lack of practical educational materials on hemostasis and coagulation testing. After developing an online educational module on coagulation for trainees, the authors observed a sustained increase in trainees’ knowledge and appropriate coagulation test ordering [1].

To address these gaps, effective teaching strategies, including chatbots [14], serious games, and objective structured clinical examination scenarios [[15], [16], [17], [18]], should be developed to improve hemostasis education. Illustrated review on common coagulation tests exists [19,20]. In a randomized controlled trial in UPC [21], a significant improvement in MD students’ results was observed when they had access to chatbots and even more so when they actually used them. Recently, in France, Perrin et al. [22] developed an adventure game called SUPER HEMO, a video game in which the player assumes the role of a protagonist in an interactive story driven by exploration and problem-solving tests. At the final hematology evaluation, MD students who played SUPER HEMO had a slightly better median score than those who did not. However, there was no significant difference for PharmD students.

Limitations include low response rates, the nonmandatory and nonrewarding nature of the survey, its size, and potential misinterpretation of questions. This survey was designed in accordance with the French MD student program for student evaluation purposes and was not intended to discriminate between physicians. The study’s single-center nature may limit generalizability. Moreover, UPC is one of France’s largest and highest-ranking medical schools.
