# Supplementary material for: Evaluation of hemostasis understanding in medical and pharmacy students from a Parisian university
Source: Res Pract Thromb Haemost. 2024 Aug 22;8(6):102547. doi: 10.1016/j.rpth.2024.102547 (PMC11414542; doi:10.1016/j.rpth.2024.102547)
Supplement: Supplementary Material [file mmc1.docx]

**Supplemental material to “Evaluation of hemostasis understanding in medical and pharmacy students from a Parisian University” by** Nicolas Gendron, Dominique Helley, Philippe Rousselot, Virginie Siguret, Pascale Gaussem, Chloé James, Lina Khider, Nadine Ajzenberg, Elodie Boissier, Nicolas Boissel, David M Smadja and Benjamin Planquette.

**Supplemental Method**

**Hemostasis Knowledge quiz**

At first, we created an educational committee composed of 2 clinical pathology experts in hemostasis (NG and DMS) and a pulmonologist specialized in thrombosis, anticoagulation and intensive care (BP). The goals of this committee were to define the pedagogical goals for the questions included in the knowledge quiz, covering various aspects of hemostasis relevant to the clinical practice of various medical specialties. The latter was designed with 5 multiple choice questions (MCQs) and 5 open-ended questions with short answers (maximum of 5 words) assessing knowledge of coagulation testing, bleeding disorders, anticoagulation therapy monitoring and reversal, as recommended in the French MD student national program led by the French College of Hematology and the College of Respiratory Medicine. MCQs and short open-ended question formats was used in this study as it is the official methods for evaluating French MD and PharmD students in France and MD and PharmD students are aware of this evaluation system during their formation.

Then, 3 professors of Hematology (DH, NA and NB) and 2 professors of Hematology (PG and VS) teaching, respectively in UPC medical and pharmaceutical schools reviewed the quiz. The feasibility and understanding of the quiz were then tested and validated by the hematology physician staff at Hôpital européen Georges Pompidou (Paris, France) to ensure that there were no errors in the corrections, no questions were difficult to understand, and the instructions were easily understandable by respondents. The quiz (**See supplemental material**) was hosted on 2 web-based platforms: i) GoogleForms for PharmD students, hematology resident physicians, vascular physicians and hemostasis consultants and ii) THEIA platform of the UPC medical school. The knowledge quiz evaluation was performed on students and hematologist residents at least 1 month after the last of hemostasis teaching. All answers were anonymized using GoogleForms. For THEIA platform, de-identification before analysis was performed to ensure that no nominative data were disseminated. The data were then stored on a secured server.

Between April and June 2022, participants performed the knowledge quiz if they agreed to participate in the study and basic demographic data (age and gender) were also collected anonymously. For MD and PharmD students the years of formation were asked and the semester of formation was asked for hematology physician residents. MD students, hematology residents and vascular physicians were asked if they performed clinical rotation in their formation in the following departments: internal medicine, vascular medicine, cardiology, intensive care unit (ICU), hematology department and hematology laboratory. The total score for the quiz was ten, with 1 point awarded for each correct question. For MCQs, answers were scored as follows: 0 discrepancies - 1 point; 1 discrepancy - 0.5 points; 2 discrepancies - 0.2 points; and more than 2 discrepancies - 0 points. Each answer of short responses was manually reviewed to identify if the answer was correct or not, according different term for a same signification. The study was conducted in accordance with the Helsinki Declaration. The study was approved by the education council and review board of the medical and pharmaceutical schools of UPC.

**Study population**

**MD students**

Our study was conducted among MD students of Paris Cité University (UPC) medical school (Paris, France). MD Students completing the 4^th^, the 5^th^ and the 6^th^ year of medicine courses in 2023, were identified since hematology is a mandatory course unit during these academic years (2nd cycle of formation of medical studies in France) before the national ranking exams and therefore the choice of the diploma of specialized studies (DSS).

**PharmD students**

Our study was conducted among PharmD students of UPC pharmaceutic school. PharmD students completing the 3^rd^ and the 4^th^ years of pharmacy courses in 2023 were identified since hematology is a mandatory course unit during these academic years (2nd cycle of formation of medical studies in France) before graduation (6^th^ year) or exams for medical residency (5^th^ year) (i.e. specialized in clinical pathology).

**Hematologist physicians**

Our study was also conducted among the French Hematologist physician’s residents and the knowledge quiz was sent to all the residents in formation for the DSS of Hematology.

**Vascular medicine physicians**

Our study was also conducted in a group of vascular medicine physicians and the knowledge quiz was sent to the member of the French club of young vascular physicians from the French Society of Vascular Medicine.

**Hemostasis consultant**

Our study was also conducted in a group of Hemostasis consultants and the knowledge quiz was sent to the member of the SFTH.

**Hemostasis Knowledge quiz**

The total score for the quiz is ten, with 1 point awarded for each correct question.

Correct answers are in bold.

- **Multiple-choice questions:**

**Scoring: 0 discrepancies - 1 point; 1 discrepancy - 0.5 points; 2 discrepancies - 0.2 points; and more than 2 discrepancies - 0 points.**

1. **An isolated, prolonged aPTT (normal PT) might suggest** (multiple answers):

A. A deficiency in Factor II

B. A deficiency in Factor V

**C. A deficiency in Factor VIII**

**D. A deficiency in Factor XI**

E. Inherited platelet disorders

1. **A prolonged aPTT with a prolonged in PT might suggest** (multiple answers):

**A. A deficiency in Factor V**

**B. Disseminated Intravascular Coagulation**

**C. Hepatocellular insufficiency**

D. A deficiency in Vitamin A

E. von Willebrand disease

1. **Regarding the monitoring of heparins at therapeutic dose** (multiple answers):

**A. Monitoring anticoagulant effect with anti-Xa activity is necessary for unfractionated heparins**

B. Monitoring anticoagulant effect with anti-Xa activity is necessary for low molecular weight heparins (LMWH).

**C. The measurement of anti-Xa activity can be done 4 hours after the injection for LMWH**

D. The anti-Xa activity of LMWH reflects their antithrombotic efficacy

E. There is no risk of heparin-induced thrombocytopenia with low molecular weight heparins

1. **Regarding anticoagulant treatments** (multiple answers):

A. Direct oral anticoagulants require regular monitoring of their anticoagulant activity

B. Direct oral anticoagulants are not contraindicated during pregnancy

C. Rivaroxaban can be administered in cases of creatinine clearance (calculated by Cockcroft and Gault formula) <30 mL/min for the treatment of venous thromboembolic disease

**D. Vitamin K antagonists require regular monitoring of their anticoagulant activity**

**E. There is an antidote for vitamin K antagonists**

1. **Regarding bleeding disorders** (multiple answers):

A. **There is no spontaneous hemorrhagic risk as long as the platelets are >30 x10^9^/L**

B. **von Willebrand disease is the most common inherited bleeding disorder**

C. Hemophilia A is usually investigated in cases of unexplained skin and mucosal bleeding

D. **Hemophilia A can be an acquired disease**

**E. Acquired platelet function disorders are more common than constitutional forms**

- **Open-ended questions with short answers**

**Scoring: 1 point for each correct question**

**Correct short answers (maximum of 5 words) are in bold.**

1. **How many hereditary thrombotic risk factors should be evaluated during thrombophilia testing?**

**5** or **Five** (deficiencies in antithrombin, protein C, protein S and mutations factor V Leiden and FIIG20210A)

1. **What is the main complication of heparin-induced thrombocytopenia?**

**Thrombosis**

1. **How many days after adjusting the dosage of a VKA treatment should the INR be checked?**

**2** and/or **3 days after**

1. **Which pathology can be ruled out with a normal D-dimer level?**

**Venous thromboembolism** (pulmonary embolism and/or deep venous thrombosis)

1. **In which situation are normal D-dimer not interpretable for the diagnosis of deep vein thrombosis and/or pulmonary embolism?**

**Under anticoagulant therapy**

**Supplemental Figure S1. Study flowchart.**

MD: medical doctor; UPC: University Paris Cité; PharmD: pharmacist; SFTH: French Society of Thrombosis and Hemostasis**.**

**
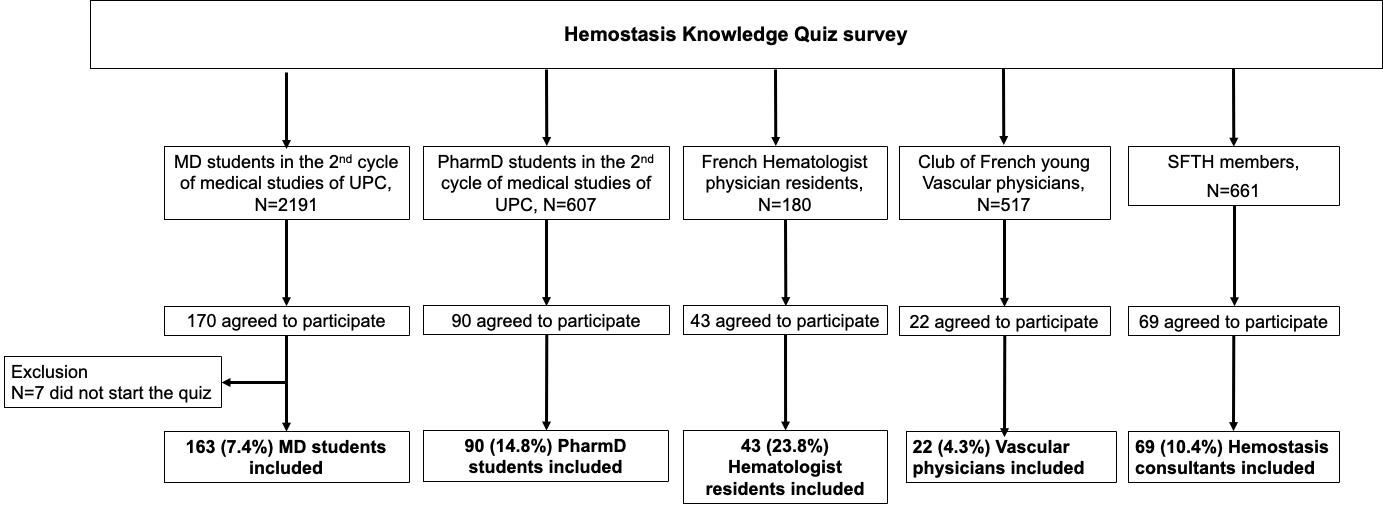
**

**Supplemental Figure S2. Correlation of hemostasis knowledge quiz score in hematologist physician residents with the number of semesters of residency.**

**Supplemental Figure S3. Hours of teaching in hemostasis and antithrombotics.**

It should be noted that PharmD students receive 3 hours of teaching in their 5^th^ year, which is after the 3^rd^ and 4^th^ years that the students included in the present study have completed. Therefore, in the present study period, MD students still have a lower total hours of hemostasis teaching during their formation when compared to PharmD students (13.33 hours versus 20.25 hours).

MD: medical doctor; PharmD: pharmacist
